# Backpropagation-Based Decoding for Multimodal Machine Translation

**DOI:** 10.3389/frai.2021.736722

**Published:** 2022-01-17

**Authors:** Ziyan Yang, Leticia Pinto-Alva, Franck Dernoncourt, Vicente Ordonez

**Affiliations:** ^1^ Department of Computer Science, University of Virginia, Charlottesville, VA, United States; ^2^ Department of Computer Science, Universidad Católica San Pablo, Arequipa, Perú; ^3^ Adobe Research, San José, CA, United States; ^4^ Department of Computer Science, Rice University, Houston, TX, United States

**Keywords:** vision and language, multimodal machine translation, backpropagation-based decoding, feedback-propagation, multimodal machine learning, computer vision, natural language processing

## Abstract

People are able to describe images using thousands of languages, but languages share only one visual world. The aim of this work is to use the learned intermediate visual representations from a deep convolutional neural network to transfer information across languages for which paired data is not available in any form. Our work proposes using backpropagation-based decoding coupled with transformer-based multilingual-multimodal language models in order to obtain translations between any languages used during training. We particularly show the capabilities of this approach in the translation of German-Japanese and Japanese-German sentence pairs, given a training data of images freely associated with text in English, German, and Japanese but for which no single image contains annotations in both Japanese and German. Moreover, we demonstrate that our approach is also generally useful in the multilingual image captioning task when sentences in a second language are available at test time. The results of our method also compare favorably in the Multi30k dataset against recently proposed methods that are also aiming to leverage images as an intermediate source of translations.

## 1 Introduction

Learning a new language is a difficult task for humans as it involves significant repetition and internalization of the association between words and concepts. People can effortlessly associate the visual stimuli of an apple sitting on top of a table with either the word “apple” in English or Êç in Chinese. However, current machine translation models usually learn these mappings between languages through large amounts of parallel multilingual text-only data. In the past few years, there have been several efforts in taking advantage of images to discover and enhance connections across different languages (Gella et al., 2017; Nakayama and Nishida, 2017; Elliott and Kádár, 2017). While some works have exploited alignments at the word-level (Bergsma and Van Durme, 2011; Hewitt et al., 2018), recent work has moved forward to finding alignments between complex sentences (Barrault et al., 2018; Surís et al., 2020; Sigurdsson et al., 2020; Yang et al., 2020).

**FIGURE 1:**
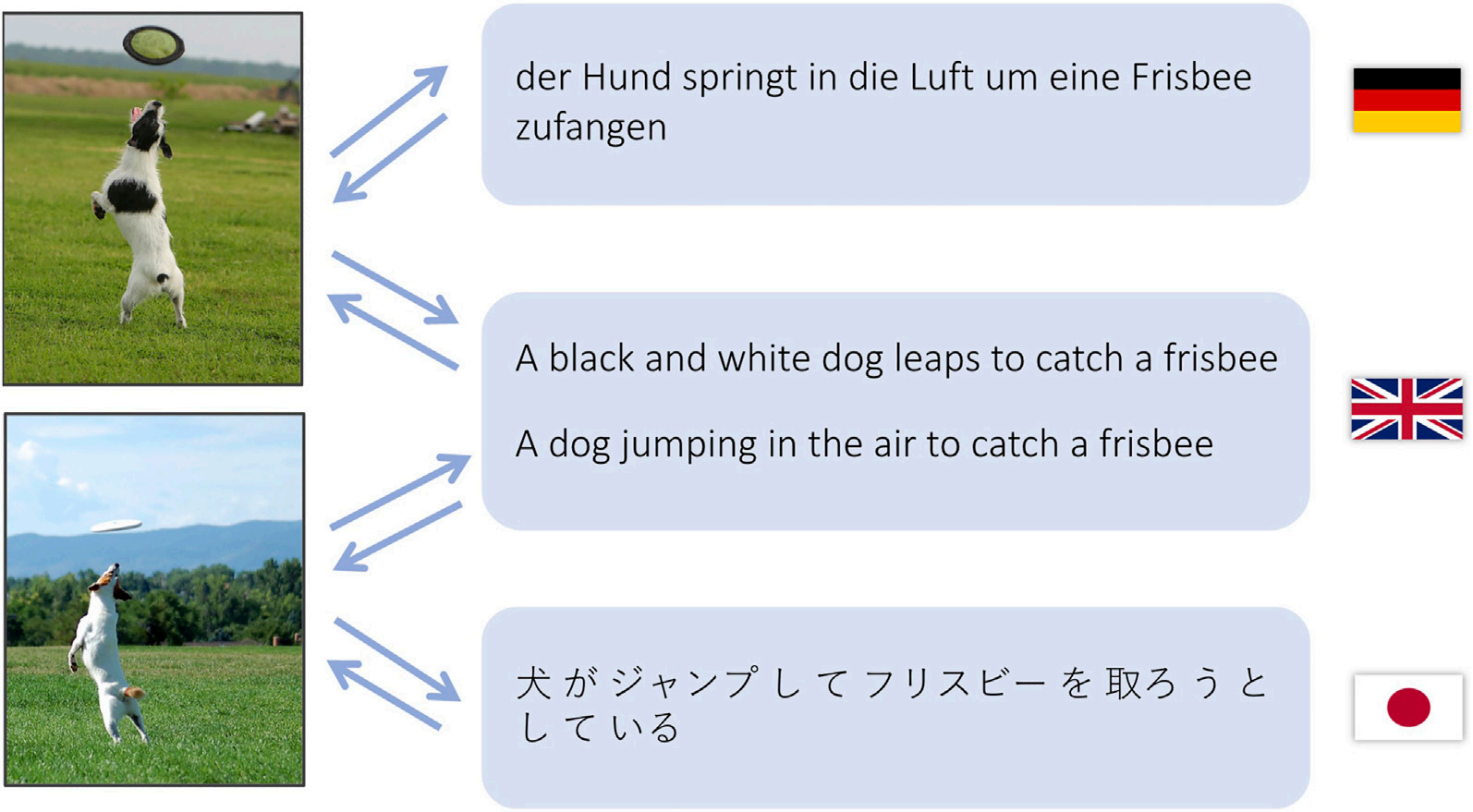
Machine translation can be done by using images as a bridge between language pairs. We show that translations between German and Japanese is possible even from a training dataset without any images annotated in both of these languages at the same time.

Multimodal machine translation aims to build word associations grounded in the visual word, however there are still some challenges. For instance, multimodal machine translation is most effective when images are provided on top of parallel text where the images enhance the traditional machine translation corpora, and a second limitation is that translation models are still required for every language pair even if there is a single common visual representation. The present work significantly extends our prior work on backpropagation-based decoding (Yang et al., 2020) using LSTMs for language pairs. Instead, we adapt transformer-based decoders for language triplets and beyond. Since our proposed approach does not train models to associate parallel texts (we do not use a language encoder), it does not require access to parallel text for any specific language pair as long as enough images with text in each target language are available. We show in Figure 1 two sample images used during training where the first image has two captions associated with it, one in German and another in English, and the second image has one caption in English and another in Japanese. Notice how the English caption for the first image is “A black and white dog leaps to catch a frisbee,” but the color of the dog is not mentioned by the German caption for the same image. Unlike some prior work in this area, our work does not assume that captions in different languages for the same image have to be translations of each other.

Our setup follows other works in natural language processing (Radford et al., 2019; Devlin et al., 2019) and vision and language (Lu et al., 2019; Chen et al., 2020; Tan and Bansal, 2019) that rely on transformer models (Vaswani et al., 2017) trained on generic datasets. However, we pretrain our model in the generic task of encoding a given input image and decoding associated sentences in several target languages. Once this model is pre-trained, we use energy-based decoding that relies on the backpropagation algorithm to use the output from a source language decoder as additional input to generate a sentence in a target language decoder. Backpropagation as a decoding mechanism has been used in some recent language generation work (Qin et al., 2020; Yang et al., 2020).

We evaluate our approach using a combination of a subset of the Multi30k dataset (Elliott et al., 2015) containing 31,014 images associated with English and German captions, and the COCO + STAIR dataset (Yoshikawa et al., 2017) containing 123,287 images associated with English and Japanese captions. Our experiments show that backpropagation-based decoding coupled with transformer-based models can produce reasonable translations among all language pairs, including for language pairs that do not share the same images. Our contributions can be summarized as: 1) Proposing a backpropagation-based decoding process using Transformers as the decoder to get machine translation results from image-text encoder-decoder models. 2) Showing that our model is effective to get translations from two languages that do not share any training image. 3) Demonstrating superior results compared to embedding training works and our earlier work on backpropagation-based decoding on recurrent neural networks.

## 2 Related Work

Our work is related to several efforts in computer vision and natural language processing. We first review image captioning methods which also rely on encoder-decoder image-to-text neural architectures. Then we discuss some representative work in unsupervised machine translation where prior work has attempted to use single modality non-parallel textual data as a source of training. Then we review two recent works on vision-language embedding methods that have used for machine translation in some capacity, that are more directly related to our work. Finally, we describe several works that have leveraged backpropagation as a decoding step during test time in the context of language models.

### 2.1 Image Captioning

Generating descriptions of images generally involves an image encoding and a text decoding step. These models are designed to recognize the contents of an input image and then use an output representation to generate understandable captions that describe the image. Vinyals et al. (2015) and Xu et al. (2015) were among a large first set of works proposing to use convolutional neural networks as the image encoder and recurrent neural networks as the text decoder to map images to text. Anderson et al. (2018) proposed to use off-the-shelf image features from an object detector, and several later works including Huang et al. (2019) and Cornia et al. (2020) follow such design. As in image captioning models, we propose training an image encoder and several text decoders, one for each language. However, while our models are trained under an image captioning objective, our final goal is finding alignments across the target languages. While most previous work on image captioning focuses on English text, recent works such as Gu et al. (2018) focused on the multilingual scenario, proposing to generate target language descriptions through pivoting language descriptions by jointly training an image captioner and an encoder-decoder translator. Unlike this previous work, our proposed approach does not use an encoder-decoder sequence-to-sequence model for machine translation, bypassing the need to train language encoders.

### 2.2 Unsupervised Machine Translation

Neural machine translation (NMT) has proposed two main directions: multilingual and multitask. For multilingual NMT, several solutions are designed to improve parameter sharing between languages. For example, Firat et al. (2016) builds one encoder and one decoder for each language, but the attention mechanism is shared by all languages. Ha et al. (2016) builds an unified approach, constructing one encoder and one decoder for all languages using language-specific coding. For multitask scenario, Luong et al. (2015) proposes to train machine translation task with other tasks such as syntactic parsing, showing quality improvement on machine translation. Anastasopoulos and Chiang (2018) combines speech transcription with machine translation, obtaining good performances on low-resource datasets. However, training machine translation models generally requires hiring professional human translators to create parallel text datasets. This expensive process of data annotation has prompted the natural language processing community to investigate unsupervised methods that do not require perfectly aligned data between source and target languages. Lample et al. (2018) uses a monolingual corpus and maps text into the same latent space; Artetxe et al. (2017) builds upon similar ideas by training shared encoders for both source and target languages; Conneau et al. (2017) learns a mapping matrix between word embeddings of different languages using nonparallel data. Pre-training on large amounts of monolingual data and conducting back translation have also demonstrated to be of importance. XLM (Lample and Conneau, 2019) and MASS (Song et al., 2019) use pre-training on a masked sequence-to-sequence task. Under the multimodal scenario, Su et al. (2019) combines visual information with encoded text information to reconstruct text input; while Huang et al. (2020) uses visual information through a back translation process. Most unsupervised machine translation systems however are still designed for training under individual bi-lingual translation tasks. Our proposed approach works does not require training on individual language pairs and does not require annotators that are fluent in more than one language.

### 2.3 Joint Visual-Language Embeddings

Recent methods that leverage images for multilingual text alignment have proposed training a joint visual-language embedding space. Sigurdsson et al. (2020) proposed to train a joint embedding space between visual data and text in different languages by leveraging instructional videos. Surís et al. (2020) proposed to jointly train a text encoder and an image encoder shared by all the visual data and all the text in 50 different languages using a contrastive loss. Our work does not explicitly train a joint visual-textual embeding space but our joint representation is induced by the captioning task and leveraged at test time by using the backpropagation algorithm to synthesize visual features from textual features.

### 2.4 Backpropagation-Based Decoding

In the training process of deep neural networks, models are optimized through backpropagation. However, backpropagation has also been used at test time for making predictions. Wang et al. (2018) designs a feedback-propagation mechanism for multi-label image classification. This work assumes partial labels are available during inference time, and uses the additional partial labels to improve the performance on other categories by backpropagating known information from the label space to update intermediate representations of the model. Qin et al. (2020) adopt a similar idea in the decoding process for a sequence prediction model. This last work achieves bi-directional sequence generation by backpropagating information from the token predicted in the current time step to the decoder so that this can potentially update the tokens predicted earlier in the sequence. Unlike prior works, our work goes further by considering the more challenging scenario where we are required to provide translations at test time for languages for which there is no image in the training data that contain annotations for both languages.

## 3 Methods

**FIGURE 2:**
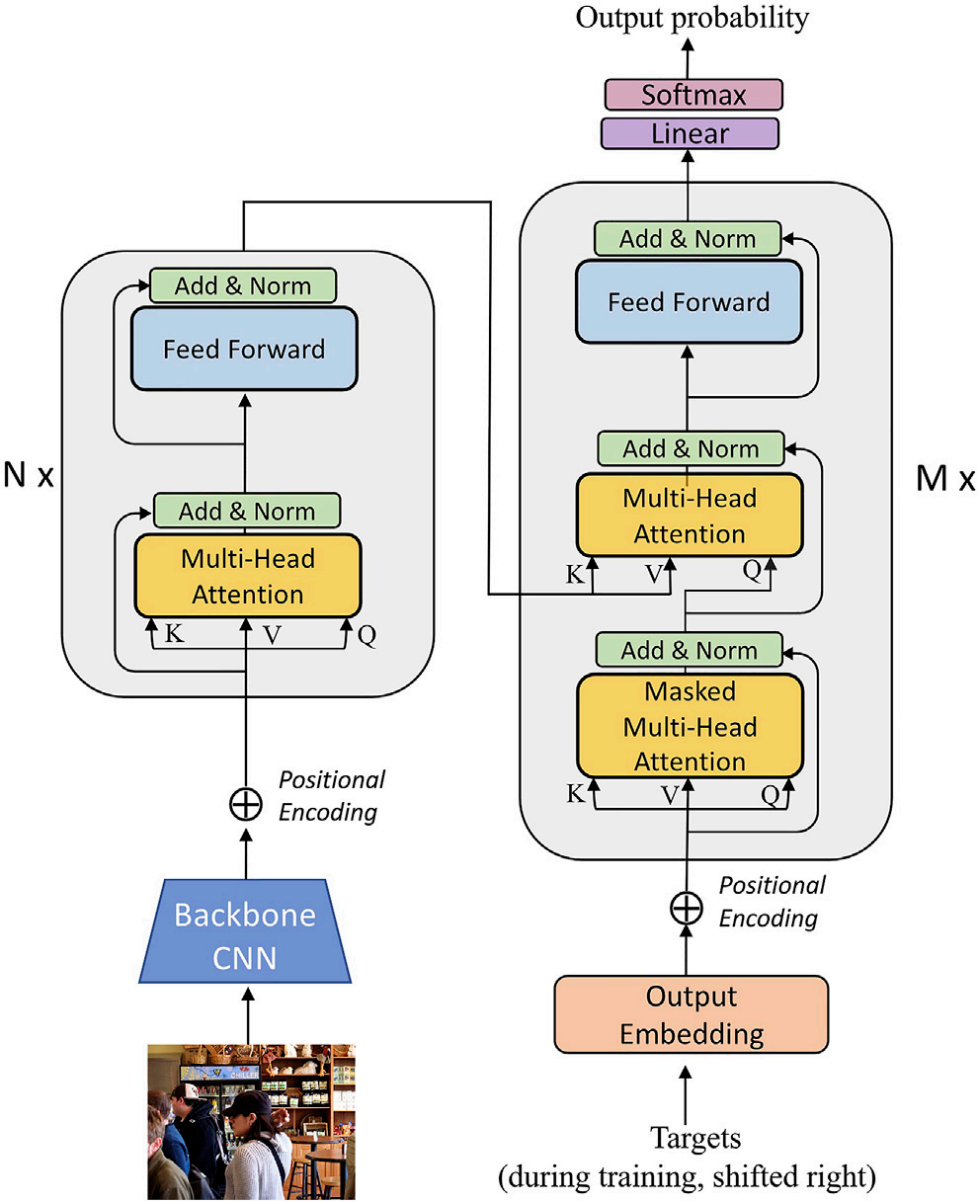
This is the detailed structure of our Transfomer-based image captioner. It adapts the original Transformer model (Vaswani et al., 2017), but inputs for the transformer encoder are obtained as image features from a pre-trained convolutional neural network.

Our approach leverages the transformer architecture as an encoder of the features obtained from a pre-trained convolutional neural network and as the language decoder for multiple languages. We will first discuss some details about combining convolutional neural networks (CNNs) and transformers to generate descriptions for images. The Transformer model proposed by Vaswani et al. (2017) was designed for the machine translation task where the input and output were text sequences. For image captioning, the input is an image instead of a sentence but the region-level feature vectors obtained from a convolutional neural network can be arranged as an input sequence of features. Figure 2 illustrates our model coupled with a single transformer-based decoder. The input images are fed into a pre-trained convolutional neural network, where the output after the last convolutional layer with adaptive pooling is represented as I ∈ R^w×h×c^. Then we reshape this image feature into I ∈ R^k×c^ where k = w × h. The feature for the whole image can be treated as a sequence of tokens, where the sequence length is k and the embedding dimension for each token is c. Similarly as in Vaswani et al. (2017), we apply positional encodings on this input sequence of image features but we adapt the formulation to 2D positional encodings. Then we feed these encoded features into a transformer encoder. We ensure that all images are the same size, therefore, when treated as a sequence of encoded features, the sequence length k is fixed.

The transformer encoder for images is similar to the one for text. It consists of *N* multi-headed self attention layers and feed-forward layers. For each attention head in a given layer, it takes a set of queries *Q*, keys *K* and values *V* as input. The output of each self-attention head is computed as $\text{softmax}\left(\frac{\text{Q}{\text{K}}^{T}}{\sqrt{d}}\right)\text{V}$ , where $\sqrt{d}$ is a normalization factor that depends on the number of dimensions *d* of the query and key vectors. In both encoder and decoder, the queries, keys, and values are the input sequences. The image encoder particularly uses the image features as the set of queries, keys, and values in the first layer. In the joint encoder-decoder layers the queries come from the decoder, and the keys and values come from the encoder layers. We follow the same setup as in Vaswani et al. (2017) and illustrate this model along with our image input in Figure 2.

In our full setup we train a single image encoder with multiple language decoders. Each language has a specific text decoder, but all of the decoders share the same convolutional neural network and transformer encoder. Let us assume we have captions that can come from three different sets: *X*, *Y*, and *Z*, each corresponding to a different language, and a set of images represented as *I*. We can define the shared image encoder as *f*(⋅) and three individual decoders for each language as *g*_*x*_(⋅), *g*_*y*_(⋅) and *g*_*z*_(⋅). Then, we can train the whole image captioning model with objective:

$$\underset{f,g_{x},g_{y},g_{z}}{\min}L_{\left(f,g_{x}\right)}\left(X,I\right)+L_{\left(f,g_{y}\right)}\left(Y,I\right)+L_{\left(f,g_{z}\right)}\left(Z,I\right).$$ (1)

**FIGURE 3:**
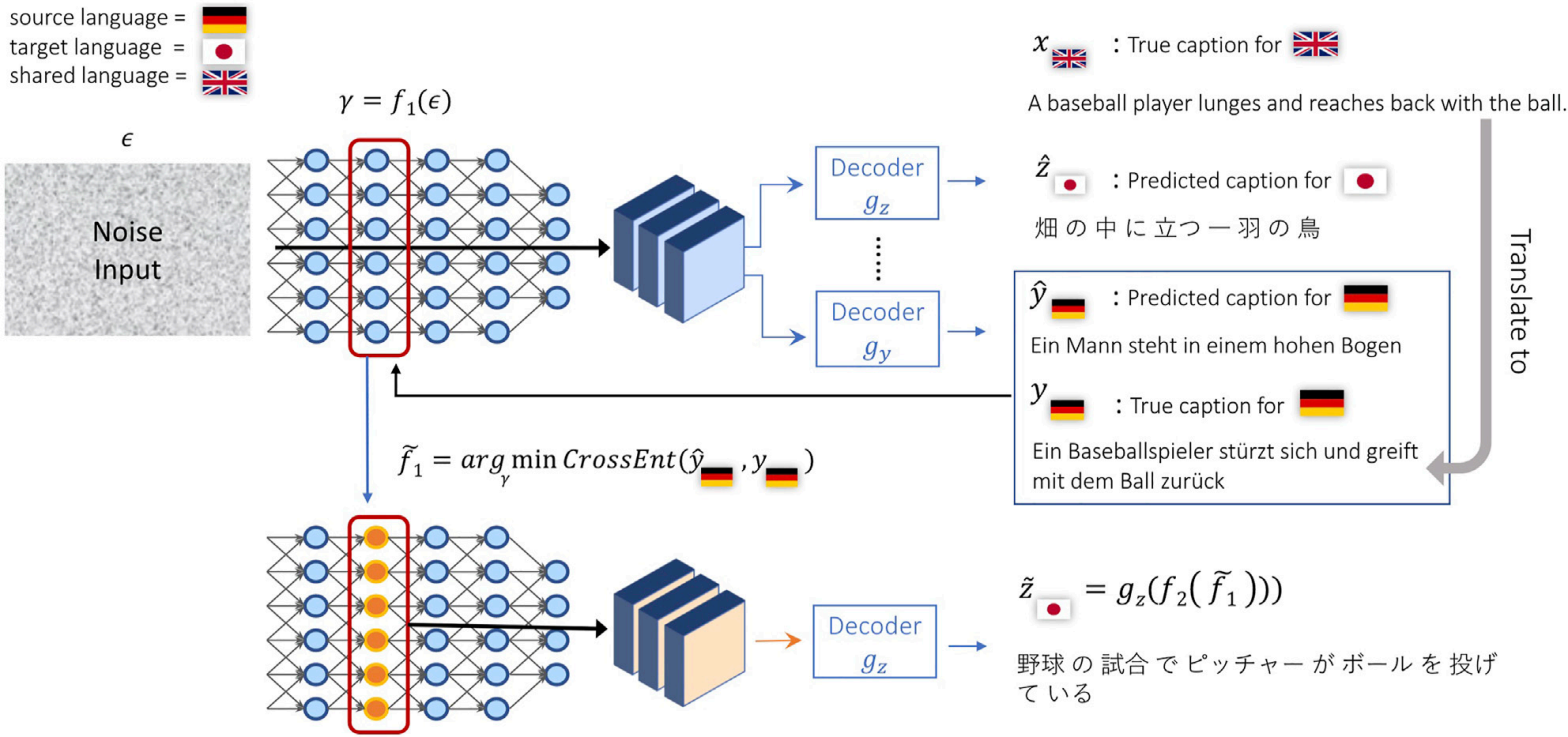
An overview of our machine translation setting where only an input text in a source language is provided. Since the model was trained as an image-to-text model, the first inference step uses noise as input to the image encoder and then generates sentences for both source and target languages. While the generated sentences from random inputs are initially meaningless, we iteratively update the intermediate image features by backpropagating information from the source caption in order to generate a meaningful sentence in the target language.

In our case, we use two bilingual datasets *D*_1_ and *D*_2_. None of them has captions for all three languages for the same image. Assume *D*_1_ has *n* images with captions in *X* and *Y*, *D*_2_ has *m* images with captions in *X* and *Z*, then we can define specifically $\left(X,I\right)={\left\{x_{i},I_{i}\right\}}_{i=1}^{i=n+m}\in D_{1}\cup D_{2}$ , $\left(Y,I\right)={\left\{y_{i},I_{i}\right\}}_{i=1}^{i=n}\in D_{1}$ and $\left(Z,I\right)={\left\{z_{i},I_{i}\right\}}_{i=1}^{i=m}\in D_{2}$ . Then, each term in Eq. 1 can be expanded as:

$$L_{\left(f,g_{x}\right)}\left(X,I\right)=L_{\left(f,g_{x}\right)}\left({\left\{x_{i},I_{i}\right\}}_{i=1}^{i=n+m}\right)=\underset{i}{\sum }\text{CrossEnt}\left(g_{x}\left(f\left(I_{i}\right)\right),x_{i}\right),$$ (2)

$$L_{\left(f,g_{y}\right)}\left(Y,I\right)=L_{\left(f,g_{y}\right)}\left({\left\{y_{i},I_{i}\right\}}_{i=1}^{i=n}\right)=\underset{i}{\sum }\text{CrossEnt}\left(g_{y}\left(f\left(I_{i}\right)\right),y_{i}\right),$$ (3)

$$L_{\left(f,g_{z}\right)}\left(Z,I\right)=L_{\left(f,g_{z}\right)}\left({\left\{z_{i},I_{i}\right\}}_{i=1}^{i=m}\right)=\underset{i}{\sum }\text{CrossEnt}\left(g_{z}\left(f\left(I_{i}\right)\right),z_{i}\right),$$ (4)

where CrossEnt(⋅,⋅) represents the cross entropy loss. Figure 3 shows our method during inference time. To define the backpropagation-based decoding, we will choose an intermediate layer in the convolutional neural network so that *f*_1_(⋅) represents the function defined by the layers before the chosen layer, and *f*_2_(⋅) represents the function defined by the layers after the chosen layer. The generated captions for the three languages can then be obtained as:

$$\hat{x_{i}}=g_{x}\left(f_{2}\left(f_{1}\left(I_{i}\right)\right)\right),$$ (5)

$$\hat{y_{i}}=g_{y}\left(f_{2}\left(f_{1}\left(I_{i}\right)\right)\right),$$ (6)

$$\hat{z_{i}}=g_{z}\left(f_{2}\left(f_{1}\left(I_{i}\right)\right)\right),$$ (7)

where the input *I*_*i*_ can be any image, and $\hat{x_{i}}$ , $\hat{y_{i}}$ , and $\hat{z_{i}}$ are the generated captions in each language. This model so far is just a regular image captioning model trained with multilingual decoders and can be used as such.

In the machine translation task however we only have an input text *x* in a source language and an output text *y* in a target language. Our goal then is to synthesize an intermediate visual feature vector $\tilde{f_{1}}\approx f_{1}\left(I\right)$ such that the text in the source language can be decoded from this input vector as $\hat{x}=g_{x}\left(f_{2}\left(\tilde{f_{1}}\right)\right)$ . We can approximate *f*_1_ iteratively by using the backpropagation algorithm to solve the following optimization setup:

$$\tilde{f_{1}}=\underset{\gamma }{\mathrm{argmin}}\underset{i}{\sum }\text{CrossEnt}\left(g_{x}\left(f_{2}\left(\gamma \right)\right),x\right),$$ (8)

where *γ* is iteratively updated until the cross-entropy loss between the predicted caption *g*_*x*_(*f*_2_(*γ*)) at the current iteration and the true caption *x* is small. The variable *γ* is initialized as *γ* = *f*_1_(*ϵ*) where *ϵ* is a dummy input image where every pixel value is sampled from a uniform distribution U(0,1) . This extends our earlier reported method (Yang et al., 2020) and demonstrates the validity of this type of approach in the trilingual scenario where one set of language pairs is not represented in the training annotations. We use a held-out set to determine the optimal amount of updates in the optimization process in Eq. 8. Once $\tilde{f_{1}}$ is obtained, then we can obtain decodings for the target language *y* as $y=g_{y}\left(f_{2}\left(\tilde{f_{1}}\right)\right)$ , and similarly for any other target language by using the corresponding language decoder.

The source and target languages can be any pair among *X*, *Y*, and *Z*, even though *Y* and *Z*, in our setup, do not share any visual information during training. One possible issue for testing our method is the lack of paired text data for *Y* and *Z*. For example, in Figure 3, *X* is English, *Y* is German and *Z* is Japanese. We are showing German as the source language and Japanese as the target language. Our datasets do not have German and Japanese captions for the same image. Therefore, we pick English as an intermediate language and translate German captions from English captions that describe the same image as Japanese captions. Since English captions and Japanese captions are created independently, the German captions translated from English captions are also independent from Japanese captions. Besides machine translation, we can also get better image captions for target languages as conducted in Yang et al. (2020) by conditioning on the source language and a given input image *I*. The only difference is during validation and test time, where *γ* is initialized as *γ* = *f*_1_(*I*) instead of using random noise as input to *f*_1_.

## 4 Experimental Setup

### 4.1 Data and Preprocessing

In order to conduct experiments and compare with previous work, we use image and multilingual text data from the benchmark datasets Multi30k (Elliott et al., 2016) and COCO + STAIR Captions (Yoshikawa et al., 2017). Multi30k includes two versions for two tasks: *Task 1* for multimodal machine translation and *Task 2* for multilingual image captioning. *Task 1* has 29,000 images for training, 1,014 images for validation and 1,000 images for testing. Each image is paired with four captions in English, German, Czech and French. These four captions are translations of each other. *Task 2* has the same image splits as *Task 1*, but each image is paired with five independently-created English captions and five independently-created German captions. As in our earlier work, we use *Task 2* data for both training and testing. We preprocess the data by tokenization, making the tokens lowercase, and selecting tokens with frequency larger than 3 to construct the vocabulary for English and German. To compare with Sigurdsson et al. (2020) and Surís et al. (2020), we train on *Task 2* but test on the testing splits for *Task 1* corresponding to German and English. We apply a Byte Pair Encoding (BPE) tokenizer and use the same vocabulary as Surís et al. (2020).

COCO + STAIR Captions include 123,287 images, where Japanese captions were collected for each image and the English captions are from the original COCO Captions Dataset (Chen et al., 2015). Each image in COCO + STAIR Captions has five independently-created English and Japanese captions. We conduct experiments on English and Japanese using this dataset. We choose to use the same split as in Karpathy and Fei-Fei (2015), which includes 113,287 images for training, 5,000 images for validation and 5,000 images for testing. To evaluate on the totally disjoint set of German and Japanese caption pairs, we combine Multi30k *Task 2* and COCO + STAIR Captions to conduct experiments.

### 4.2 Implementation

We provide here some concrete details about our implementation of the model described earlier. The number of transformer encoder layers is 2 and the number of transformer decoder layers is 4, both with eight heads. The backbone image encoder is Resnet-50 (He et al., 2016) pre-trained on ImageNet’s large scale visual recognition challenge classification task (Deng et al., 2009). We have separate decoders for different languages, but all the decoders share the same image encoder and transformer encoder. We fine-tune both image encoder and the transformer encoder and decoders during training with learning rate 4e-4. During inference time, we use Conv-40 as the pivoting layer, as this proved to be most effective on a held out set. All of our code and experimental setup will be distributed using a public repository to ensure reproducibility upon publication and code is included in this submission as supplementary material. The datasets used in this project are all public datasets, Multi30k and COCO + STAIR.

## 5 Results and Discussion

For this section, we will discuss our results under two settings: first, we compare our method with Yang et al. (2020) and adapt to the same settings on real image inputs and noise image inputs on Multi30k *Task2* and COCO + STAIR to evaluate on English-German English-Japanese and German-Japanese language pairs; then, we follow the settings of Surís et al. (2020) using Multi30k *Task1 + Task2* data to show the machine translation results.

### 5.1 Bilingual Results

First, we conduct experiments on Multi30k *Task 2* for multi-lingual captioning and translation in German and English. We compare our results against a baseline using LSTMs as decoders while keeping the vocabulary and convolutional neural network encoder the same. Table 1 reports results under three conditions: 1) the model takes only images as inputs without additional information (standard image captioning); 2) the model takes images as inputs and uses additional captions as “partial evidence” (Wang et al., 2018) to improve the caption in a second language (image captioning with partial evidence); and 3) the model takes as input only text from a source language to generate text for a target language (machine translation). Our method shows better results in all the conditions for all the metrics compared to the LSTM baseline as described in our earlier work in Yang et al. (2020).

**TABLE 1:** Results on Multi30k with German and English unpaired textual captions for training and testing under three task setups: standard image captioning, image captioning under partial evidence, and machine translation.

Decoder	Input	Target	BLEU-4	ROUGE-L	CIDEr
LSTM	Image	German	16.08	46.53	43.13
	Image	English	24.40	50.94	51.30
	Image + English	German	22.21	52.12	59.63
	Image + German	English	28.15	54.06	61.53
	English	German	17.24	48.65	44.67
	German	English	20.82	49.71	44.57
Transformer	Image	German	16.51	47.02	45.26
	Image	English	25.18	51.47	53.21
	Image + English	German	23.45	53.32	65.76
	Image + German	English	29.13	55.13	66.49
	English	German	17.84	49.32	48.26
	German	English	22.88	51.29	51.76

Next, we report in Table 2 experimental results on COCO-STAIR Captions for Japanese and English. We report results on the *karpathy testing split* consisting of 5,000 images on this dataset. As in our prior experiment we compare against a strong baseline of LSTM-based decoders under the same three conditions: standard image captioning, image captioning under partial evidence, and machine translation. We observe that sometimes the best model for image captioning does not necessarily lead to the best model for decoding under the backpropagation-based decoding process. For instance, as can be observed in Table 2, the LSTM-based image captioner obtains 32.31 points in BLEU score while the Transformer-based image captioner obtains 30.70 points in BLEU score under the same conditions. However, the Transformer-based decoder is more effective under the backpropagation-based decoding needed for the machine translation task (22.76 BLEU) compared to the LSTM-based decoder (21.70 BLEU). Therefore, when we select models for testing, we also consider the performance using backpropagation-based decoding as a stopping criteria since it is not our goal in this work to obtain the best image captioning model but the most useful model for pivoting across languages. We keep the model selection criterion the same for both models. As a consequence, the selected Transformer-based model does not outperform the LSTM-based model in image captioning (when taking only images as inputs), but the backpropagation decoding works better for the Transformer-based model in both conditional image captioning and multimodal machine translation.

**TABLE 2:** Results on COCO + STAIR with Japanese and English unpaired textual captions for training and testing under three task setups: standard image captioning, image captioning under partial evidence, and machine translation.

Decoder	Input	Target	BLEU-4	ROUGE-L	CIDEr
LSTM	Image	Japanese	37.71	57.13	92.26
	Image	English	32.31	53.79	99.62
	Image + English	Japanese	40.26	58.57	100.71
	Image + Japanese	English	34.34	55.31	107.71
	English	Japanese	29.51	51.59	68.78
	Japanese	English	21.70	45.10	67.43
Transformer	Image	Japanese	37.74	57.08	91.67
	Image	English	30.70	52.77	95.08
	Image + English	Japanese	40.87	58.91	102.87
	Image + Japanese	English	33.12	54.78	106.87
	English	Japanese	31.10	52.60	76.40
	Japanese	English	22.76	46.80	75.82

### 5.2 Trilingual Results

Next we demonstrate how our proposed method can be easily extended to multilingual cases using multiple datasets. We particularly evaluate our method on two disjoint languages, which means these two languages do not have any parallel corpora or common set of images annotated in both languages. In this case, we train a trilingual image captioning model for English, German, and Japanese caption generation using both Multi30k *Task 2* and COCO + STAIR Captions. This model consists of one shared image encoder and three independent decoders for three languages: English, German, and Japanese. During training, every batch samples the same number of images from Multi30k *Task 2* and COCO + STAIR Captions, then the corresponding captions are used to train the model end-to-end. At test time, since we need German captions for COCO + STAIR Captions and Japanese captions for Multi30k *Task 2* in order to evaluate the image captioning task under partial evidence, and the machine translation task, we use Google Translate to generate translations from English captions in these two datasets. While these are not ground truth, they are of sufficient quality to assess the extent of the potential improvements obtained with our method over baseline accuracies.

**FIGURE 4:**
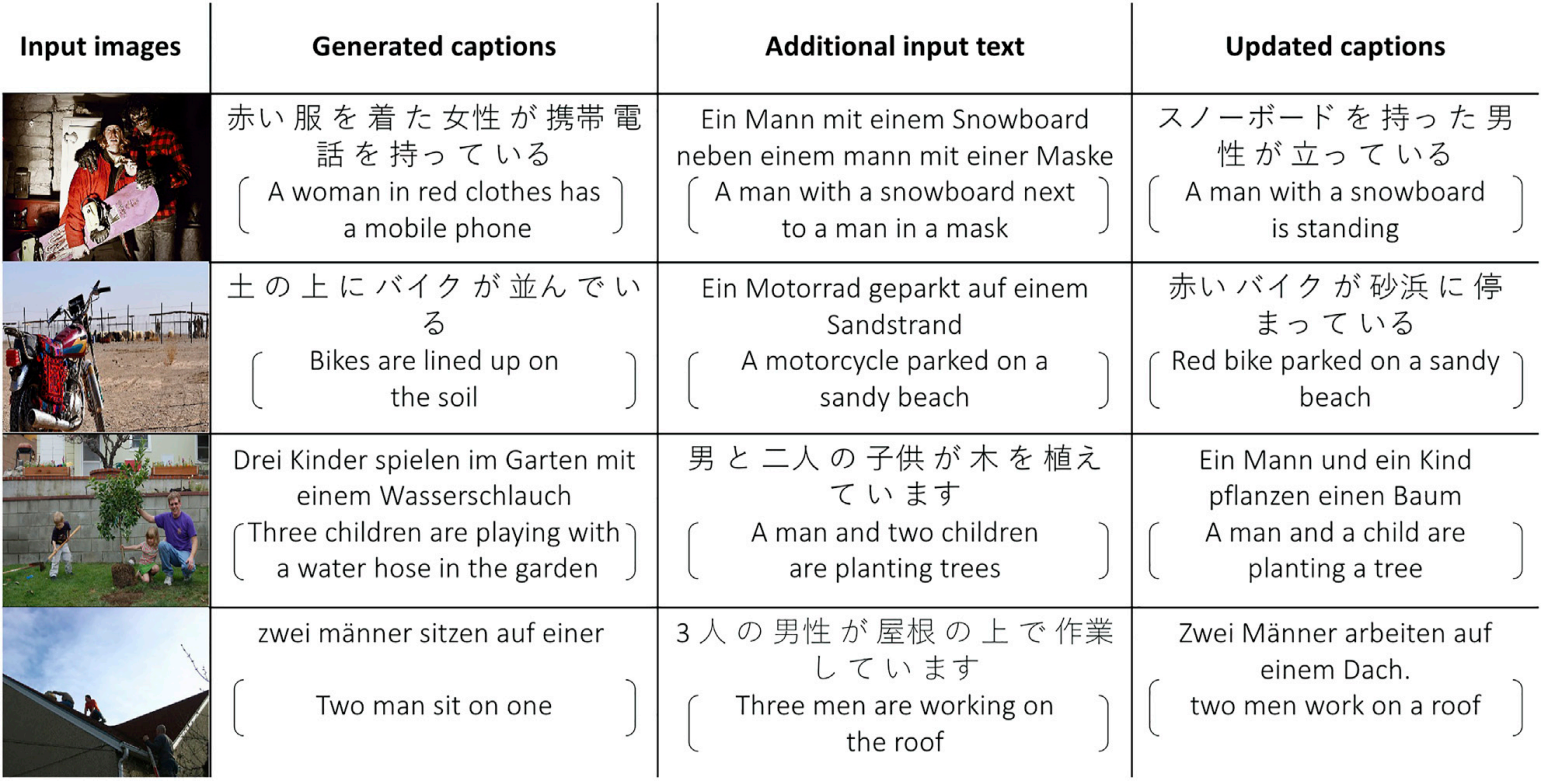
We show some examples of using additional source language information to improve target language caption generation. The first column shows the input images, the second column shows generated captions conditioned only on the input images, the third column shows ground truth text for that image in either German or Japanese, the fourth and last column shows the predicted caption conditioned both on the input image (first column) and the additional input text (third column). English sentences obtained with Google Translate are provided for reference inside the brackets.

We report the results on Table 3 The improvement from baseline image caption generation to conditional image captioning is still consistent even though the provided source language captions are translations from another language (English). Transformer-based models outperform LSTM-based models in most cases, but in the case of using only German captions to generate Japanese and using only Japanese captions to generate German, the LSTM-based model and Transformer-based model demonstrate similar results. German captions and Japanese captions in the training process do not share any images, but during inference, German and Japanese text can still help each other to generate better captions. Qualitative examples from the Transformer-based model are provided in Figure 4, for German and Japanese. For example, in the first instance, the generated Japanese caption (second column) incorrectly identified the gender of the subject as “女性” which translates as “female.” However, after we provide the German caption “Ein Mann mit einem Snowboard neben einem Mann mit einer Maske” to the decoder, then the gender is identified correctly. These results show the possibility to apply our method in a more general situation: during training, the image captioner is trained on several independent monolingual image captioning datasets, and the image features can be used as a shared feature space to transfer information among languages, even for languages for which no available bilingual annotators can be found, as long as people can be instructed to annotate images, translations can be obtained.

**TABLE 3:** Results on Multi30k *Task2* and COCO + STAIR with Japanese and German unpaired textual captions.

Decoder	Input	Target	BLEU-4	ROUGE-L	CIDEr
LSTM	Image	Japanese	36.62	56.31	88.51
	Image	German	16.28	46.58	43.90
	Image + German	Japanese	37.45	56.76	91.41
	Image + Japanese	German	17.92	48.15	47.75
	German	Japanese	17.36	43.19	31.16
	Japanese	German	8.38	39.45	19.30
Transformer	Image	Japanese	37.76	57.24	91.50
	Image	German	16.64	46.79	46.78
	Image + German	Japanese	39.18	57.93	95.80
	Image + Japanese	German	19.80	49.14	53.18
	German	Japanese	16.69	42.30	31.68
	Japanese	German	9.45	39.16	22.47

**FIGURE 5:**
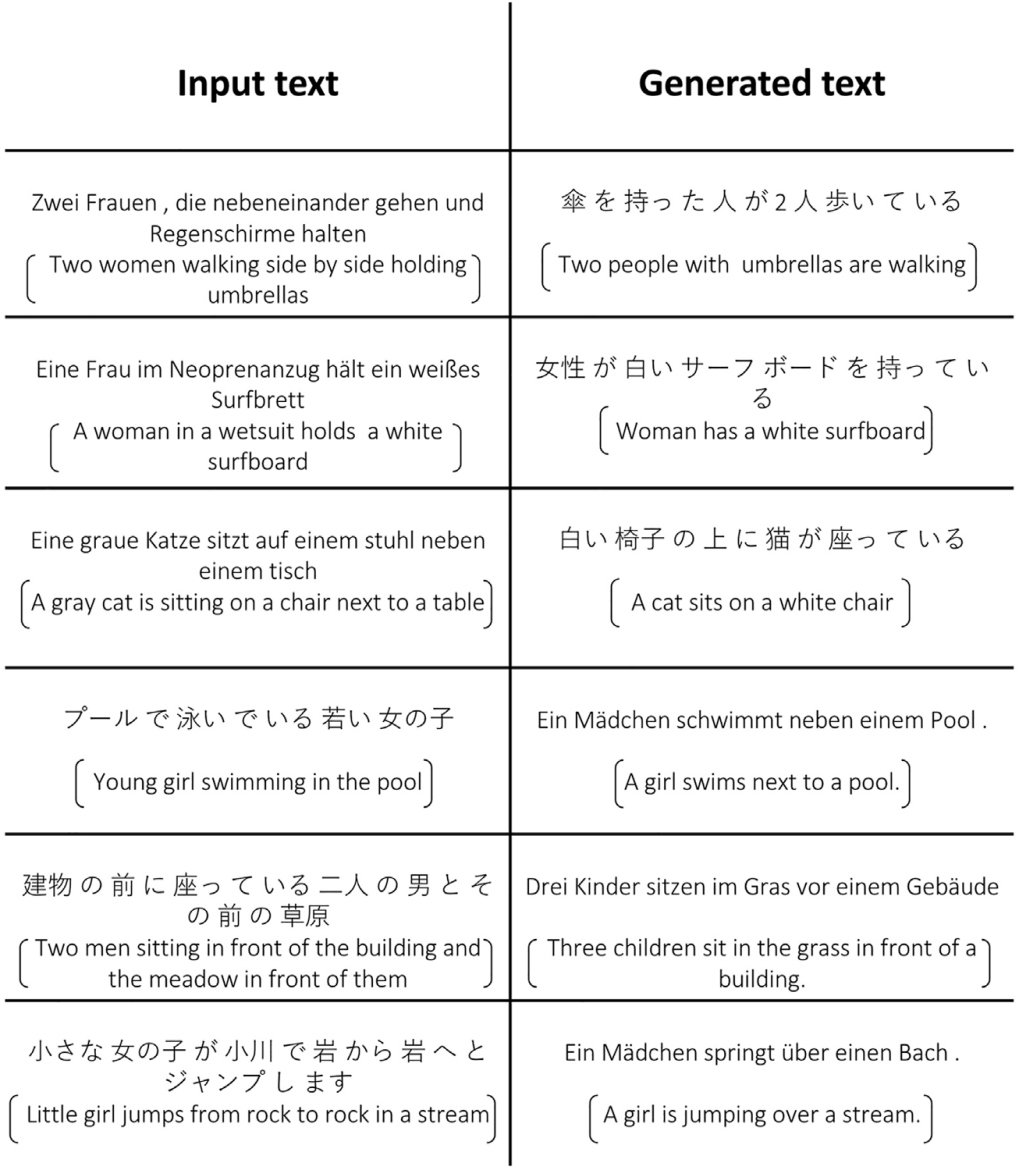
We show some examples of German-Japanese translation. English sentences by Google Translate are provided for reference inside the brackets.

For the multimodal machine translation case, we show results in Figure 5. Even though the model was not trained to align text pairs, it can still provide reasonable translations by synthesizing visual features using the source language text. For example, in the first row of Figure 5, it is hard for the model to recognize the gender of the subjects in the image without visual information, but significant information in tokens such as “傘”(umbrella) and “歩い”(walking) are successfully translated. There are other details lost such as the color of the referred cat in the example on the third row “eine graue Katze,” which is omitted in the generated Japanese caption which only generates the token for cat (猫). This happens because the model works by taking the input sentence in German and then “imagining” the visual features of an image through the backpropagation-based decoding process, and then translating these “imagined” visual features into Japanese text. Some information is lost in the process regarding gender which is impractical to obtain from an image or other challenging attributes but important actions and objects are often preserved.

### 5.3 Visual Grounding Results

Recent state-of-the-art works using visual information to align languages include V-Grounding (Sigurdsson et al., 2020) and Globetrotter (Surís et al., 2020). V-Grounding aligns languages by learning a joint visual-language embedding space. This work constructs separate text embeddings for different languages, and one shared encoder for visual information. Globetrotter learns cross-lingual representations by leveraging alignments between text in multiple languages and images. They build one text encoder and one image encoder that are shared by all the languages and images. Both works do not need parallel multilingual data during training, instead, they use paired information between images and languages. To compare with previous works, we use Multi30k *Task2* English and German caption data for training and *Task1* translation data for inference. We split 29,000 training images into 14,500/14,500 for English and German, using disjoint image sets and text data to train an image captioning model. We follow the experimental settings of Surís et al. (2020), using the same Byte Pair Encoding (BPE) tokenization (Sennrich et al., 2015) to construct a vocabulary, and train our image captioning model with transformers as decoder. During inference time, Multi30k *Task1* data includes 1,014 images for validation and 1,000 images for testing. Each image is paired with one English caption and one German caption, and these captions are translations of each other. We generate translations for the target language by feeding back source language sentences using backpropagation-based decoding.

V-Grounding (Sigurdsson et al., 2020) and Globetrotter (Surís et al., 2020) try to project text and visual information to a common joint embedding space, and these works cannot do sentence generation directly. Therefore, we extract text features of source language sentences using their pre-trained embedding space and retrieve the most similar target language sentences from the training set. We use the models provided by Surís et al. (2020), which are pre-trained using 50 languages and fine-tuned on English captions using COCO, Flickr30k (Plummer et al., 2015) and the Google Conceptual Captions dataset (Sharma et al., 2018). In Table 4 we show that our method outperforms these competing methods under all metrics (BLEU-4, ROUGE, and CIDEr) in both the English to German and German to English translation pairs. In general, German to English gets higher scores than English to German for all the models–as German is generally considered morphologically rich and less configurational (Fraser et al., 2013).

**TABLE 4:** Results on Multi30k *Task1* English (EN) to German (DE) and German (DE) to English (EN) translation tasks, comparing with previous vision-language joint embedding works.

Method	Source-Target	BLEU-4	ROUGE-L	CIDEr
Sigurdsson et al. (2020)	English to German	2.25	15.88	18.96
	German to English	5.01	23.90	28.71
Globetrotter Surís et al. (2020)	English to German	3.94	19.31	31.74
	German to English	6.00	23.68	37.36
LSTMs Yang et al. (2020)	English to German	5.46	25.12	40.28
	German to English	11.73	33.75	70.62
Ours	English to German	8.41	30.40	61.71
	German to English	15.02	38.93	96.37

## 6 Conclusion

Our work shows that under backpropagation-based decoding we are able to synthesize visual features from text in a source language in order to decode text in a target language, thus obtaining text translations even if the model was not trained for translation. We demonstrated this capability for the first time in a trilingual setting where two of the languages did not have any pairings, as in no image in the training data contained annotations in both of these languages. Our results show a path to build multimodal and multilingual pre-trained models that can implicitly learn alignments among languages. This is especially relevant for low resource languages for which paired data is not available due to lack of translators for that language but for which monolingual native speakers can be found and directed to annotate images instead of text. Our code is available as supplementary material and will be released publicly upon publication to ensure reproducibility.

## Data Availability

Publicly available datasets were analyzed in this study. This data can be found here: https://github.com/multi30k/dataset, https://stair-lab-cit.github.io/STAIR-captions-web/.

## References

[B1] Anastasopoulos A. Chiang D. (2018). Tied Multitask Learning for Neural Speech Translation. arXiv preprint arXiv:1802.06655

[B2] Anderson P. He X. Buehler C. Teney D. Johnson M. Gould S. (2018). “Bottom-up and Top-Down Attention for Image Captioning and Visual Question Answering,” in Proceedings of the IEEE conference on computer vision and pattern recognition, 6077–6086. 10.1109/cvpr.2018.00636

[B3] Artetxe M. Labaka G. Agirre E. Cho K. (2017). Unsupervised Neural Machine Translation. arXiv preprint arXiv:1710.11041.

[B4] Barrault L. Bougares F. Specia L. Lala C. Elliott D. Frank S. (2018). “Findings of the Third Shared Task on Multimodal Machine Translation,” in Proceedings of the Third Conference on Machine Translation (WMT ’18). 10.18653/v1/w18-6402

[B5] Bergsma S. Van Durme B. (2011). “Learning Bilingual Lexicons Using the Visual Similarity of Labeled Web Images,” in IJCAI Proceedings-International Joint Conference on Artificial Intelligence (Citeseer), 1764.22

[B6] Chen X. Fang H. Lin T.-Y. Vedantam R. Gupta S. Dollár P. (2015). Microsoft Coco Captions: Data Collection and Evaluation Server. arXiv preprint arXiv:1504.00325

[B7] Chen Y.-C. Li L. Yu L. El Kholy A. Ahmed F. Gan Z. (2020). “Uniter: Universal Image-Text Representation Learning,” in European Conference on Computer Vision (Springer), 104–120. 10.1007/978-3-030-58577-8_7

[B8] Conneau A. Lample G. Ranzato M. Denoyer L. Jégou H. (2017). Word Translation without Parallel Data. arXiv preprint arXiv:1710.04087

[B9] Cornia M. Stefanini M. Baraldi L. Cucchiara R. (2020). “Meshed-memory Transformer for Image Captioning,” in Proceedings of the IEEE/CVF Conference on Computer Vision and Pattern Recognition, 10578–10587. 10.1109/cvpr42600.2020.01059

[B10] Deng J. Dong W. Socher R. Li L.-J. Li K. Fei-Fei L. (2009). “Imagenet: A Large-Scale Hierarchical Image Database,” in 2009 IEEE conference on computer vision and pattern recognition (Ieee), 248–255. 10.1109/cvpr.2009.5206848

[B11] Devlin J. Chang M.-W. Lee K. Toutanova K. (2019). “Bert: Pre-training of Deep Bidirectional Transformers for Language Understanding,” in Proceedings of the 2019 Conference of the North American Chapter of the Association for Computational Linguistics: Human Language Technologies, 4171–4186. Long and Short Papers.Vol. 1

[B12] Elliott D. Frank S. Hasler E. (2015). Multi-language Image Description with Neural Sequence Models. CoRR. abs/1510.04709 3.

[B13] Elliott D. Frank S. Sima’an K. Specia L. (2016). “Multi30k: Multilingual English-German Image Descriptions,” in Proceedings of the 5th Workshop on Vision and Language. 10.18653/v1/w16-3210

[B14] Elliott D. Kádár Á. (2017). “Imagination Improves Multimodal Translation,” in Proceedings of the Eighth International Joint Conference on Natural Language Processing (Volume 1: Long Papers), 130–141.

[B15] Firat O. Cho K. Bengio Y. (2016). Multi-way, Multilingual Neural Machine Translation with a Shared Attention Mechanism. arXiv preprint arXiv:1601.01073. 10.18653/v1/n16-1101

[B16] Fraser A. Schmid H. Farkas R. Wang R. Schütze H. (2013). Knowledge Sources for Constituent Parsing of German, a Morphologically Rich and Less-Configurational Language. Comput. Linguistics 39, 57–85. 10.1162/coli_a_00135

[B17] Gella S. Sennrich R. Keller F. Lapata M. (2017). “Image Pivoting for Learning Multilingual Multimodal Representations,” in Proceedings of the 2017 Conference on Empirical Methods in Natural Language Processing, Copenhagen, Denmark (Copenhagen: Association for Computational Linguistics), 2839–2845. 10.18653/v1/D17-1303

[B18] Gu J. Joty S. Cai J. Wang G. (2018). “Unpaired Image Captioning by Language Pivoting,” in The European Conference on Computer Vision (ECCV). 10.1007/978-3-030-01246-5_31

[B19] Ha T.-L. Niehues J. Waibel A. (2016). Toward Multilingual Neural Machine Translation with Universal Encoder and Decoder. arXiv preprint arXiv:1611.04798

[B20] He K. Zhang X. Ren S. Sun J. (2016). “Deep Residual Learning for Image Recognition,” in Proceedings of the IEEE Conference on Computer Vision and Pattern Recognition, 770–778. 10.1109/cvpr.2016.90

[B21] Hewitt J. Ippolito D. Callahan B. Kriz R. Wijaya D. T. Callison-Burch C. (2018). “Learning Translations *via* Images with a Massively Multilingual Image Dataset,” in Proceedings of the 56th Annual Meeting of the Association for Computational Linguistics (Volume 1: Long Papers), Melbourne, Australia (Melbourne: Association for Computational Linguistics). 10.18653/v1/p18-1239

[B22] Huang L. Wang W. Chen J. Wei X.-Y. (2019). “Attention on Attention for Image Captioning,” in Proceedings of the IEEE/CVF International Conference on Computer Vision, 4634–4643. 10.1109/iccv.2019.00473

[B23] Huang P.-Y. Hu J. Chang X. Hauptmann A. (2020). Unsupervised Multimodal Neural Machine Translation with Pseudo Visual Pivoting. arXiv preprint arXiv:2005.03119

[B24] Karpathy A. Fei-Fei L. (2015). “Deep Visual-Semantic Alignments for Generating Image Descriptions,” in Proceedings of the IEEE conference on computer vision and pattern recognition, 3128–3137. 10.1109/cvpr.2015.7298932 27514036

[B25] Lample G. Conneau A. (2019). Cross-lingual Language Model Pretraining. arXiv preprint arXiv:1901.07291

[B26] Lample G. Conneau A. Denoyer L. Ranzato M. (2018). “Unsupervised Machine Translation Using Monolingual Corpora Only,” in International Conference on Learning Representations.

[B27] Lu J. Batra D. Parikh D. Lee S. (2019). “Vilbert: Pretraining Task-Agnostic Visiolinguistic Representations for Vision-And-Language Tasks,” in Advances in Neural Information Processing Systems. Editors Wallach H. Larochelle H. Beygelzimer A. d’ Alché-Buc F. Fox E. Garnett R. (Vancouver, Canada: Curran Associates, Inc.), Vol. 32.

[B28] Luong M.-T. Le Q. V. Sutskever I. Vinyals O. Kaiser L. (2015). Multi-task Sequence to Sequence Learning. arXiv preprint arXiv:1511.06114

[B29] Nakayama H. Nishida N. (2017). Zero-resource Machine Translation by Multimodal Encoder–Decoder Network with Multimedia Pivot. Machine Translation 31, 49–64. 10.1007/s10590-017-9197-z

[B30] Plummer B. A. Wang L. Cervantes C. M. Caicedo J. C. Hockenmaier J. Lazebnik S. (2015). “Flickr30k Entities: Collecting Region-To-Phrase Correspondences for Richer Image-To-Sentence Models,” in Proceedings of the IEEE international conference on computer vision, 2641–2649. 10.1109/iccv.2015.303

[B31] Qin L. Shwartz V. West P. Bhagavatula C. Hwang J. D. Le Bras R. (2020). “Backpropagation-based Decoding for Unsupervised Counterfactual and Abductive Reasoning,” in Proceedings of the 2020 Conference on Empirical Methods in Natural Language Processing (EMNLP), 794–805.

[B32] Radford A. Wu J. Child R. Luan D. Amodei D. Sutskever I. (2019). Language Models Are Unsupervised Multitask Learners.

[B33] Sennrich R. Haddow B. Birch A. (2015). Neural Machine Translation of Rare Words with Subword Units. arXiv preprint arXiv:1508.07909

[B34] Sharma P. Ding N. Goodman S. Soricut R. (2018). “Conceptual Captions: A Cleaned, Hypernymed, Image Alt-Text Dataset for Automatic Image Captioning,” in Proceedings of ACL. 10.18653/v1/p18-1238

[B35] Sigurdsson G. A. Alayrac J.-B. Nematzadeh A. Smaira L. Malinowski M. Carreira J. (2020). Visual Grounding in Video for Unsupervised Word Translation. In Proceedings of the IEEE/CVF Conference on Computer Vision and Pattern Recognition. 10850–10859. 10.1109/cvpr42600.2020.01086

[B36] Song K. Tan X. Qin T. Lu J. Liu T.-Y. (2019). “Mass: Masked Sequence to Sequence Pre-training for Language Generation,” in International Conference on Machine Learning, 5926–5936.

[B37] Su Y. Fan K. Bach N. Kuo C.-C. J. Huang F. (2019). “Unsupervised Multi-Modal Neural Machine Translation,” in Proceedings of the IEEE Conference on Computer Vision and Pattern Recognition, 10482–10491. 10.1109/cvpr.2019.01073

[B38] Surís D. Epstein D. Vondrick C. (2020). Globetrotter: Unsupervised Multilingual Translation from Visual Alignment. arXiv preprint arXiv:2012.04631

[B39] Tan H. Bansal M. (2019). “Lxmert: Learning Cross-Modality Encoder Representations from Transformers,” in Proceedings of the 2019 Conference on Empirical Methods in Natural Language Processing and the 9th International Joint Conference on Natural Language Processing (EMNLP-IJCNLP), 5103–5114. 10.18653/v1/d19-1514

[B40] Vaswani A. Shazeer N. Parmar N. Uszkoreit J. Jones L. Gomez A. N. (2017), Attention Is All You Need. Advances in Neural Information Processing Systems, 5998–6008.

[B41] Vinyals O. Toshev A. Bengio S. Erhan D. (2015). “Show and Tell: A Neural Image Caption Generator,” in Proceedings of the IEEE conference on computer vision and pattern recognition, 3156–3164. 10.1109/cvpr.2015.7298935

[B42] Wang T. Yamaguchi K. Ordonez V. (2018). “Feedback-prop: Convolutional Neural Network Inference under Partial Evidence,” in Proceedings of the IEEE Conference on Computer Vision and Pattern Recognition, 898–907. 10.1109/cvpr.2018.00100

[B43] Xu K. Ba J. Kiros R. Cho K. Courville A. Salakhudinov R. (2015). “Show, Attend and Tell: Neural Image Caption Generation with Visual Attention,” in International Conference on Machine Learning, 2048–2057.

[B44] Yang Z. Pinto-Alva L. Dernoncourt F. Ordonez V. (2020). “Using Visual Feature Space as a Pivot across Languages,” in Proceedings of the 2020 Conference on Empirical Methods in Natural Language Processing: Findings, 3673–3678. 10.18653/v1/2020.findings-emnlp.328

[B45] Yoshikawa Y. Shigeto Y. Takeuchi A. (2017). “STAIR Captions: Constructing a Large-Scale Japanese Image Caption Dataset,” in Proceedings of the 55th Annual Meeting of the Association for Computational Linguistics (Volume 2: Short Papers) (Vancouver, Canada: Association for Computational Linguistics), 417–421. 10.18653/v1/p17-2066

